# A phase II trial of capecitabine (Xeloda®) in recurrent ovarian cancer

**DOI:** 10.1038/sj.bjc.6601381

**Published:** 2003-11-11

**Authors:** P A Vasey, L McMahon, J Paul, N Reed, S B Kaye

**Affiliations:** 1 Beatson Oncology Centre, Western Infirmary, Glasgow G11 6NT, UK; 2 The Royal Marsden Hospital, Downs Road, Sutton SM2 5PT, UK

**Keywords:** capecitabine, fluoropyrimidine, ovarian cancer, quality of life

## Abstract

Oral capecitabine is a highly active, well-tolerated and convenient treatment for breast and colorectal cancer. This trial assessed the efficacy and safety of single-agent capecitabine in patients with previously treated ovarian cancer. A total of 29 patients with platinum-pretreated relapsed ovarian cancer were enrolled in this prospective, open-label, single-centre, phase II study. Patients received oral capecitabine 1250 mg m^−2^ twice daily on days 1–14 of a 21-day cycle. Tumour response was evaluated using serum CA125. Out of 29 enrolled patients, 28 were evaluable, and a response was observed in eight patients (29%, 95% confidence interval (CI), 13–49%). Median progression-free and overall survivals were 3.7 (95% CI, 2.8–4.6) and 8.0 (95% CI, 4.1–11.8) months, respectively. After 6 months of treatment, 28% (95% CI, 13–48%) of patients remained progression-free and 62% (95% CI, 42–79%) were still alive. The most common clinical adverse events were hand–foot syndrome (HFS), nausea and diarrhoea. Grade 3 HFS occurred in 14% of patients, grade 3 vomiting in 10%. Efficacy and safety of capecitabine compare favourably with other monotherapies in platinum-refractory epithelial ovarian cancer. The convenience and improved safety profile of capecitabine compared with intravenous. regimens make it an ideal agent for administration in the outpatient setting.

Despite the development of more effective chemotherapy and the refinement of surgical techniques, ovarian cancer remains the number one cause of death from gynaecological cancer in the Western world (Jemal *et al*, 2002). The treatment of ovarian cancer has become characterised by high response rates to first-line therapies, followed by relapse in the majority of patients and generally poor results in the second-line setting. Platinum-based regimens have consistently achieved response rates of around 70% when used as first-line therapy (Thigpen, 2000), but the high rate of relapse has meant that 5-year survival in patients with ovarian cancer remains around 50% (Ozols, 2002).

The most important predictor of response to second-line treatment is the time from initial chemotherapy, known as the treatment-free interval. This has been best characterised for platinum (Blackledge *et al*, 1989; Gore *et al*, 1990; Markman *et al*, 1991), where a spectrum of sensitivity to rechallenge with platinum agents has been observed. Patients relapsing more than 24 months after initial platinum therapy have a 60% or greater chance of a further response, whereas patients relapsing within 6 months may have as little as a 10–15% chance of a further response with these agents. Although several agents, including anthracyclines, taxanes, gemcitabine and topotecan, have been used in patients with platinum-pretreated ovarian cancer, there is a general paucity of randomised trial data in this setting (Markman, 2002).

Many patients with advanced ovarian cancer have disease that cannot be adequately visualised using CT scans or ultrasonography, and CA125 response has been shown to be an effective and satisfactory way of evaluating biological activity. An analysis of 19 clinical trials evaluating 14 different drugs in relapsed ovarian cancer demonstrated that definitions of response, based on both a 50% and 75% decrease of CA125 concentrations, accurately predicted which drugs were active and justified further investigation (Rustin *et al*, 2000). In these studies, the CA125 and clinical response criteria were concordant in 20 of the 25 treatment groups, including docetaxel, gemcitabine, oxaliplatin, paclitaxel and topotecan. Response rates according to CA125 are slightly higher than standard clinical response rates, but based on the analysis above, Rustin *et al* (2000) concluded that response rates were so similar that either standard or CA125 response criteria could be used in clinical trials.

The goal of second-line therapy in ovarian cancer is essentially palliative, and therefore factors such as patient convenience and comfort, toxicity profile and cost are primary concerns in drug selection in this setting (Gadducci *et al*, 2001). Accordingly, health-related quality of life (QOL) assessment has become increasingly important in gynaecological oncology (Pignata *et al*, 2001). For this reason, effective treatment options that do not compromise tolerability or patients' convenience are desirable.

5-Fluorouracil (5-FU) modulated by folinic acid has shown promise in the treatment of patients with platinum-refractory ovarian cancer. In a study evaluating bolus 5-FU administered at a dose of 370 mg m^−2^ with folinic acid 200 mg m^−2^ for 5 consecutive days every 4–5 weeks, a 17% response rate was seen and the regimen was generally well tolerated (Look *et al*, 1995). The highly active, oral fluoropyrimidine capecitabine (Xeloda®: F Hoffmann-La Roche, Basel, Switzerland) was rationally developed to deliver 5-FU preferentially to tumours by exploiting the high concentrations of thymidine phosphorylase in tumour tissue. Capecitabine is absorbed through the intestinal mucosa as an intact molecule and is converted to 5-FU via a three-step enzymatic activation cascade, involving thymidine phosphorylase and cytidine deaminase, both of which are upregulated in ovarian cancer cells (Miwa *et al*, 1998).

Capecitabine showed promise in phase I trials that included patients with ovarian cancer (Budman *et al*, 1998; Mackean *et al*, 1998), and a wide-ranging clinical trial programme continues to examine its efficacy and safety in a number of malignancies. Following impressive results in phase III trials, capecitabine has become established as an effective and well-tolerated first-line therapy for patients with advanced colorectal cancer (Hoff *et al*, 2001; Van Cutsem *et al*, 2001; Twelves, 2002). Based on results from extensive clinical trials in more than 700 patients with metastatic breast cancer (MBC) (Blum *et al*, 1999; Blum *et al*, 2001; Reichardt *et al*, 2001; Fumoleau *et al*, 2002; Miller *et al*, 2002), capecitabine monotherapy is considered by many to be the reference treatment in taxane-pretreated MBC and is the only agent to have received worldwide regulatory approval in this setting. In addition, the combination of capecitabine plus docetaxel 75 mg m^−2^ has been shown to significantly improve survival in anthracycline-pretreated patients with MBC, with a median 3-month survival benefit compared with docetaxel 100 mg m^−2^ alone (O'Shaughnessy *et al*, 2002). This combination has consequently received regulatory approval in more than 50 countries as therapy for anthracycline-pretreated patients with MBC.

Recent studies have demonstrated a strong patient preference for oral rather than intravenous (i.v.) therapies, with two independent studies showing a preference for oral therapy in 84 and 89% of patients, as long as efficacy is not sacrificed (Liu *et al*, 1997; Borner *et al*, 2002). A third study demonstrated that home-based therapy results in significant improvements in QOL compared with hospital-based therapy in the palliative setting (Payne, 1992).

We conducted an open-label, phase II trial of single-agent capecitabine in patients with previously treated ovarian cancer to assess the efficacy and safety of this regimen, and to determine its impact on QOL.

## PATIENTS AND METHODS

### Study design

This prospective, open-label, single-centre, phase II study examined the efficacy and safety of capecitabine in women with platinum-pretreated epithelial ovarian cancer. The study was approved by the local ethics committee (West Ethics Committee, North Glasgow Trust, UK) and run in accordance with the Declaration of Helsinki. All patients provided signed, informed consent before study entry.

### Eligibility criteria

Women with platinum-pretreated epithelial ovarian cancer that had relapsed within 12 months of the last course of chemotherapy were eligible for the study. Patients were required to have previously received a platinum agent, although not necessarily as part of the most recent treatment regimen. A maximum of three previous lines of chemotherapy was allowed. Patients were required to have measurable disease, defined as serum CA125⩾100 kU l^−1^, with documented CA125 progression (Rustin *et al*, 1996). Other eligibility criteria included age⩾18 years, performance status <3 (ECOG scale), life expectancy⩾18 weeks and adequate bone marrow (neutrophils >2 × 10^9^ l^−1^, platelets >75 × 10^9^ l^−1^), liver (AST and/or ALT <5 times upper limit of normal) and renal (serum creatinine <1.5 times upper limit of normal) function. Patients with clinically significant cardiac disease or who had experienced myocardial infarction within the previous 12 months were excluded, as were those who had evidence of CNS metastases, or known sensitivity or prior severe reaction to 5-FU.

### Treatment schedule

The treatment schedule consisted of oral capecitabine 1250 mg m^−2^ administered twice daily for 14 days, followed by a 7-day rest period. Treatment was administered orally within 30 min of breakfast and dinner, and swallowed with approximately 200 ml of water. The cycle was repeated every 21 days and all patients received at least two cycles of study treatment. Dose interruption and reduction were used to manage adverse events and the scheme is summarised in Table 1

**Table 1:** Capecitabine dose modification scheme

	**Grade 2**	**Grade 3**	**Grade 4**
First appearance	Interrupt treatment until resolved to grade 0–1, then continue at same dose with prophylaxis^a^ where possible	Interrupt treatment until resolved to grade 0–1, then continue at 75% dose, with prophylaxis^a^where possible	Interrupt treatment unless the investigator considers it to be in the best interests of the patient to continue at 50% dose once toxicity has resolved to grade 0–1
Second appearance	Interrupt treatment until resolved to grade 0–1, then continue at 75% dose	Interrupt treatment until resolved to grade 0–1, then continue at 50% dose	
Third appearance	Interrupt treatment until resolved to grade 0–1, then continue at 50% dose	Discontinue treatment – off study	
Fourth appearance	Discontinue treatment – off study		

a For example, loperamide for diarrhoea, emollients and pyridoxine for hand–foot syndrome.

. Patients responding or stable after 18 weeks could continue on treatment indefinitely at the investigator's discretion, until disease progression or intolerable toxicity.

### Assessment of response

The primary end points of this phase II study were response rate and duration of response, determined using weekly measurements of serum CA125. Response was defined as fulfilling either of the following criteria: (A) if there was a 50% decrease in serum CA125 from two previous, consistently elevated samples; or (B) if there has been a serial decrease in CA125 concentrations of more than 75% over three samples (Rustin *et al*, 1996). In both these definitions, the final sample must have been taken at least 28 days after the previous sample. In patients with clinically or radiologically evaluable disease at baseline, specific tumour assessments including CT scans and/or measurements were carried out at 6-weekly intervals and response was defined by modified Southwest Oncology Group (SWOG) response criteria.

Safety assessments were performed at each clinic visit. Adverse events were monitored throughout the study period and for 28 days after stopping study treatment. Patients were advised to contact their oncology team if they experienced symptoms possibly related to treatment. Adverse events were graded according to National Cancer Institute Common Toxicity Criteria (NCI CTC). Haematological assessments, including full blood count and differential, were carried out at each patient visit. Liver and renal functions were assessed on a 3-weekly basis and parameters included AST/ALT, alkaline phosphatase, serum protein, total bilirubin, urea and electrolytes and serum creatinine. Chest X-rays were performed if clinically indicated.

The impact of therapy or disease progression/evolution on patients' well-being was assessed by self-administration of the European Organisation for Research and Treatment of Cancer (EORTC) Quality of Life questionnaires (QLQ-C30 version 3 and QLQ-C24 version 1). Patients were required to complete these questionnaires at baseline and prior to each 3-weekly cycle.

### Statistical methods

A two-stage Gehan design was employed with a target response rate of 20%, power set at 95% and a precision of 10%. This design requires a maximum of 25 evaluable patients. Overall survival and progression-free survival were estimated using the Kaplan–Meier method. All times were measured from the start of treatment.

## RESULTS

Recruitment began in May 1999, and 29 patients had been entered by study close (extra patients were recruited to allow for unevaluables) in November 2001. By February 2002, median follow-up duration from the start of treatment for living patients was 16 months (range 8–28 months). Patients had received a median of two lines of chemotherapy, 13 (45%) had undergone surgery prior to study enrolment and three had received prior radiotherapy. Patient characteristics and treatment histories are summarised in Table 2

**Table 2:** Patient characteristics and treatment histories

No. of patients	29
Median age: range (years)	57 (38–78)
*Performance status*	
0	8 (28%)
1	17 (59%)
2	1 (3%)
Unknown	3 (10%)
	
*Disease stage at diagnosis*	
Ic	1 (3%)
II	1 (3%)
III	17 (59%)
IV	10 (34%)
	
*Degree of differentiation at diagnosis*	
Moderate	7 (24%)
Poor	22 (76%)
	
*Prior chemotherapy: number of courses*	
1	4 (14%)
2	13 (45%)
>2	12 (41%)
	
*Treatment-free interval*	
>6 months	8 (28%)
<6 months	21 (72%)
	
*Prior platinum chemotherapy: best response*	
Complete/partial response	20 (70%)
Stable disease	3 (10%)
Progressive disease	0
Not evaluable/unknown	6 (21%)
	
*Platinum-free interval*	
>6 months	20 (70%)
<6 months	9 (30%)

NB=no patients had received 5-FU previously.

. No patients had previously received 5-FU-based chemotherapy. Median CA125 at study entry was 884 kU l^−1^ (range 153–9750), and the median platinum-free interval was 8 months (range 2–47).

A total of 121 cycles of capecitabine were administered, with patients receiving a median of four (range 1–9) cycles. Seven patients required dose reductions, of whom two required a second dose reduction. Six cycles were delayed because of treatment-related adverse events. Four patients omitted capecitabine doses on several days because of treatment-related adverse events (two grade 3 hand–foot syndrome (HFS), one grade 3 diarrhoea, one grade 3 vomiting). Treatment-related adverse events occurred early in the treatment course and were readily managed with dose interruption and, if necessary, dose reduction. Two patients withdrew from treatment because of toxicity (grade 3 HFS in both cases).

### Responses and survival

The CA125 response was observed in eight of 28 evaluable patients giving a response rate of 29% (95% confidence interval (CI), 13–49%). One patient was unevaluable because of insufficient CA125 concentrations. There were no significant differences in response rates according to platinum-free interval <6 or>6 months (44 *vs* 21%, *P*=0.47) or treatment-free interval <6 or >6 months (24 *vs* 33%), but the small patient numbers in this comparison make identification of small differences difficult.

**Figure 1:**
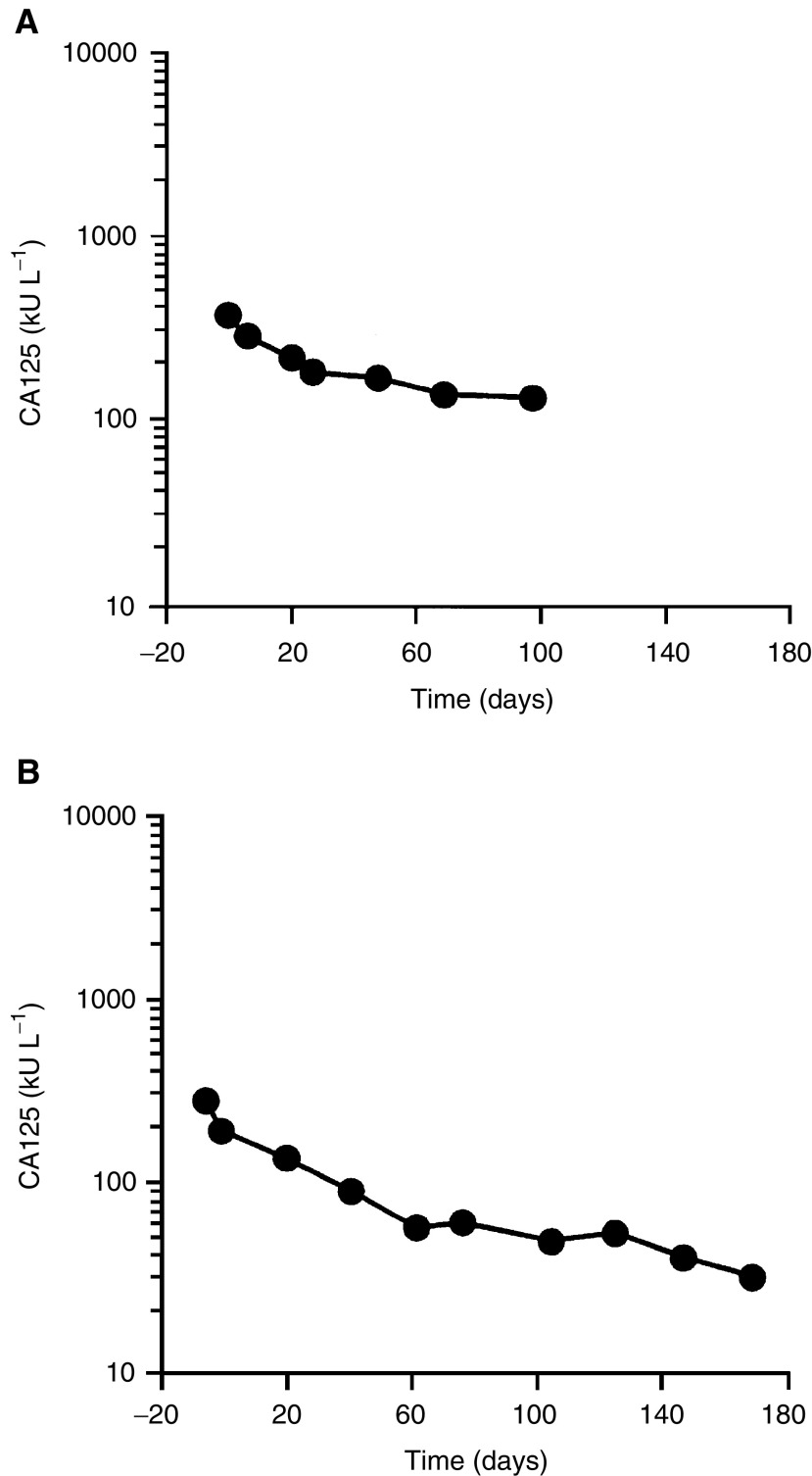
Typical responder profiles (CA125 response curves).

Typical responder profiles for two patients are illustrated in Figure 1

**Figure 2:**
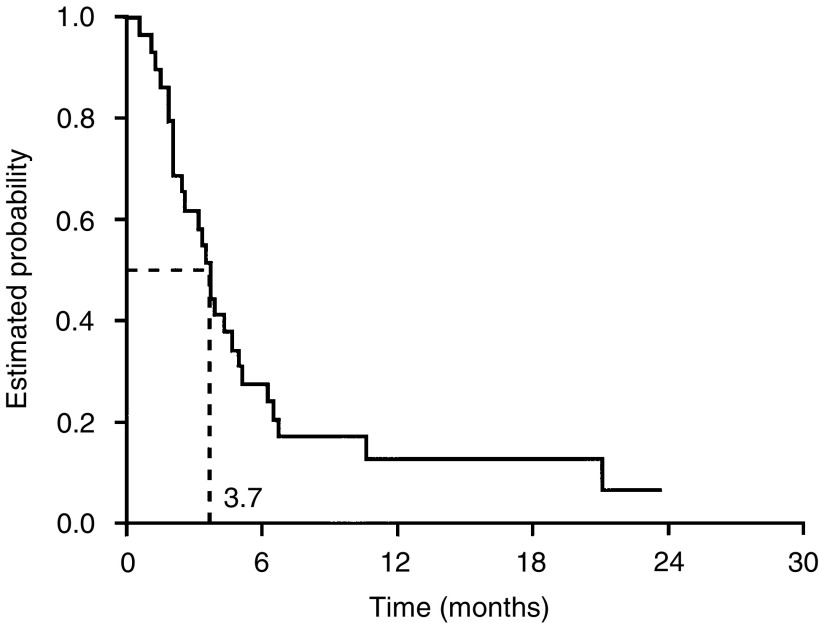
Progression-free survival.

. Median progression-free and overall survival durations were 3.7 (95% CI, 2.8–4.6) months and 8.0 (95% CI, 4.1–11.8) months, respectively (Figures 2

and 3

**Figure 3:**
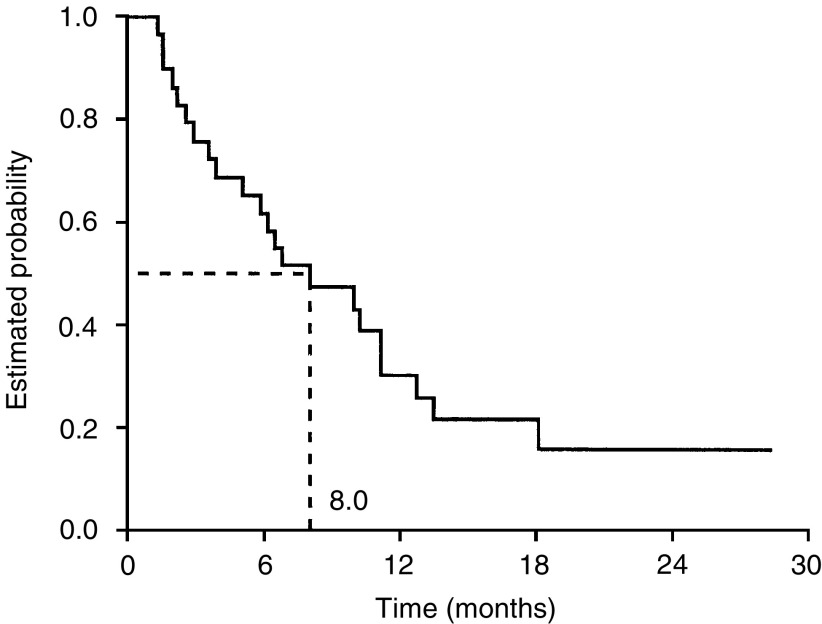
Overall survival.

). After 6 months of treatment, 28% (95% CI, 13–48%) of patients remained progression-free and 62% (95% CI, 42–79%) were still alive.

Drainage of ascites can affect the CA125 dynamics, although there are no robust guidelines about how to interpret this. It has been suggested that values taken within 4 weeks of this procedure should be discounted (GJS Rustin, personal communication). Six patients in the study had ascites drained either during the treatment period, or up to 3 weeks before. If the CA125 values collected for these patients within 4 weeks of drainage are ignored, response rate is reduced to 27%, as two patients would be unable to be classified as responders from the remaining values.

In total, 14 patients had measurable disease at baseline, and a complete radiological response was observed in one patient, giving a clinical overall response rate of 7%. This patient was not evaluable for the CA125 response despite having a sustained >50% fall in this marker, because one of her values was >10% higher than the previous value during the study. Disease stabilisation occurred in a further five patients, resulting in an overall rate of disease control of 43% according to radiological assessment.

### Toxicity summary

All 29 patients were evaluable for safety (Table 3

**Table 3:** Most common (⩾10%) treatment-related adverse events, all grades (*n*=29)

	**All grades**	**Grade 3**	**Grade 4**
**Adverse event**	**No (%)**	**No (%)**	**No (%)**
Anaemia	23 (79)	1 (3)	0
Hand–foot syndrome	18 (62)	4 (14)	0
Nausea	17 (59)	1 (3)	0
Diarrhoea	17 (59)	2 (7)	0
Vomiting	14 (48)	3 (10)	0
Leucopenia	13 (45)	0	1 (3)
Fatigue	12 (41)	1 (3)	0
Neutropenia	9 (31)	1 (3)	0
Stomatitis	9 (31)	0	0
Thrombocytopenia	8 (28)	0	0
Constipation	6 (21)	0	0
Anorexia	3 (10)	1 (3)	0

). Capecitabine was very well tolerated, with the most common clinical adverse events being HFS, nausea and diarrhoea. The only grade 3 adverse event occurring in more than 10% of patients was grade 3 HFS, a non-life-threatening side effect. This occurred in four patients (14%), and grade 3 vomiting occurred in three patients (10%). Myelosuppression and alopecia were rare, with only one patient experiencing grade 3 neutropenia and one patient experiencing mild alopecia. The only grade 4 adverse event was uncomplicated leucopenia, which occurred in one patient. There were no treatment-related deaths during the study.

### Health-related QOL

The number of patients evaluable for QOL in each cycle was as follows: 29 (cycle 1); 28 (cycle 2); 23 (cycle 3); 18 (cycle 4); 12 (cycle 5); 5 (cycle 6). Of the 29 patients, 28 (97%) evaluable for QOL completed their questionnaires prior to cycle 1 and 75−80% of patients completed questionnaires prior to cycles 2−6.

When averaged over available assessments, mean change in QOL scores from baseline indicated a significant decline in physical function together with an increase in levels of fatigue during the course of chemotherapy (*P*=0.021 and 0.049, respectively). There was a trend towards significant reduction in role function (*P*=0.054), but in other domains of the questionnaire, no significant decreases in averaged scores were noted.

## DISCUSSION

Capecitabine is an active agent for the treatment of patients with ovarian cancer previously treated with platinum and taxanes. Using CA125 concentrations to indicate response, capecitabine resulted in a response rate of 29% in a population of 28 evaluable patients. Capecitabine also demonstrated a favourable and manageable safety profile in this trial, with a particularly low rate of myelosuppression and alopecia. The most common grade 3 clinical adverse event was the cutaneous disorder HFS, which is never directly life-threatening and is readily managed using dose modification and/or treatment interruption. Grade 3 HFS occurred in 14% of patients and 10% experienced grade 3 vomiting. A similar safety profile was observed during extensive phase II and III trials of capecitabine in MBC and metastatic colorectal cancer (Cassidy *et al*, 2002; O'Shaughnessy, 2002). The only previous experience with this agent in recurrent ovarian cancer demonstrated a clinical response rate of 25% (one complete response and two partial responses in 12 evaluable patients) using the same doses but administered on a 28-day schedule (Boehmer and Jaeger, 2002).

The interpretation of the CA125 response in patients who have undergone ascitic drainage is difficult. There are a number of different strategies that may be employed in this situation, including the exclusion from the analysis of all patients who have had ascites drained, or using predetermined criteria based on the frequency of evaluable CA125 measurements during treatment. In our study, three of the eight patients with the CA125 response had required drainage of ascites. In one patient, ascitic drainage was performed after observation of CA125 response, but in the remaining two patients, one of whom had two drainage procedures performed, three of 14 and six of 10 of their CA125 values were taken within 4 weeks of drainage of ascites. Following exclusion of these values, neither patient would be considered to be a responder, despite clearly showing clinical benefit from capecitabine treatment. Further investigation of the effect of ascitic drainage on interpretation of the CA125 response dynamics is required.

The intrinsic variability of CA125 estimations needs to be addressed, particularly with reference to the frequency of sampling. This is particularly relevant in this study, because the one patient with measurable disease who had a clinical response could not be classified as a CA125 responder. A single weekly CA125 value was >10% higher than the previous result, and therefore that patient is not classified as responding despite having a sustained >50% reduction from baseline.

As CA125 sampling is far less expensive than multiple CT scanning, it may lead to great cost savings for health-care systems. Many patients with relapsed ovarian cancer do not have clinically or radiologically measurable disease, and the use of CA125 response as the primary outcome measure consequently allows the inclusion of more patients in clinical trials. Providing patients have an initial CA125 concentration that is high enough to observe the requisite 50% fall, more patients will be eligible for entry into such trials.

The efficacy and safety of capecitabine in this setting compare favourably with other available monotherapies, including topotecan, paclitaxel and pegylated liposomal doxorubicin (PLD). Although the numbers of patients in this study are small, there is evidence that capecitabine is active against refractory tumours. Large, randomised trials have compared topotecan with paclitaxel or PLD in patients progressing after first-line platinum-based therapy. In these studies, response rates of 17–20.5% for topotecan, 13% for paclitaxel and 20% for PLD were observed (ten Bokkel Huinink *et al*, 1997; Gordon *et al*, 2001). In our study, a response rate of 29% was achieved in a relatively heavily pretreated population of patients (median of two previous chemotherapies). The median progression-free survival (3.7 months) achieved in our study is similar to those of topotecan (3.7–5.3 months), paclitaxel (3.2 months) and PLD (3.7 months).

One of the main advantages of capecitabine over other available therapies is its convenience and tolerability. In contrast to agents such as topotecan and paclitaxel (ten Bokkel Huinink *et al*, 1997), grade 3 neutropenia was rare, and observed in only one patient during our study and there was only one grade 4 event (leucopenia). In addition, alopecia occurred in only one (3%) patient treated with capecitabine, at grade 1 intensity.

High attrition rates hinder the interpretation of the QOL data collected during the study, in addition to the lack of an observation-only comparator. Averaged scores showed a statistically significant decline in physical function and increase in fatigue over the course of the study, but only one patient experienced grade 3 fatigue. QOL scores in other domains, including social function and body image, were not significantly reduced. Moreover, cognitive function, body image and attitude to disease/treatment showed trends towards improved QOL. In other studies, the addition of capecitabine to docetaxel significantly improved survival without compromising quality of life in 255 patients (Twelves *et al*, 2001). There is increasing evidence that patients' judgements of their QOL are quite accurate and consistent (Waldron *et al*, 1999); nevertheless, the sensitivity of the EORTC questionnaires to the benefits of an oral treatment is not proven. Ongoing studies of capecitabine are using convenience and satisfaction questionnaires (FACIT) and will improve our understanding of the effect of patients' values, preferences and life priorities on treatment decisions.

The potential for capecitabine as the backbone of effective combination regimens has been established in numerous studies in breast, colorectal and gastrointestinal malignancies. In these indications, capecitabine in combination with other cytotoxic agents, including docetaxel, oxaliplatin and irinotecan, has demonstrated high activity and an acceptable safety profile (Kerr *et al*, 2002; O'Shaughnessy *et al*, 2002; Sastre *et al*, 2002). Although combination regimens have yet to demonstrate benefits in efficacy compared with single-agent therapy in randomised trials of patients with relapsed ovarian cancer (Chi and Sabbatini, 2000), this should not automatically preclude trials investigating new combinations that may achieve an acceptable balance of efficacy and tolerability. Indeed, the highest response rates in phase II studies of platinum-resistant disease can be seen using dose-dense combination regimens (Meyer *et al*, 2001; van der Burg *et al*, 2002). The potential for capecitabine to be used in combination with agents that upregulate thymidine phosphorylase in relapsed ovarian cancer is clear.

In summary, capecitabine has demonstrated promising activity and a favourable safety profile in the treatment of platinum-refractory epithelial ovarian cancer. This agent is now being investigated further in patients with relapsed ovarian cancer. The safety and convenience advantages afforded to patients over current i.v. options make capecitabine an ideal agent for administration in the outpatient setting, potentially freeing them from the burden of i.v. therapy.
